# Utilization of Artificial Intelligence in Disease Prevention: Diagnosis, Treatment, and Implications for the Healthcare Workforce

**DOI:** 10.3390/healthcare10040608

**Published:** 2022-03-24

**Authors:** Shahid Ud Din Wani, Nisar Ahmad Khan, Gaurav Thakur, Surya Prakash Gautam, Mohammad Ali, Prawez Alam, Sultan Alshehri, Mohammed M. Ghoneim, Faiyaz Shakeel

**Affiliations:** 1 Department of Pharmaceutical Sciences, University of Kashmir, Jammu and Kashmir, Srinagar 190006, India; nkhan2008@gmail.com; 2 Department of Pharmaceutics, CT Institute of Pharmaceutical Sciences, CT Group of Institutions, Jalandhar 144020, India; thakurg058@gmail.com (G.T.); gautamsuryaprakash@gmail.com (S.P.G.); 3 Department of Pharmacology, School of Pharmaceutical Sciences, University of Science & Technology, Meghalaya 793101, India; alimohammad973@gmail.com; 4 Department of Pharmacognosy, College of Pharmacy, Prince Sattam Bin Abdulaziz University, Al-Kharj 11942, Saudi Arabia; prawez_pharma@yahoo.com; 5 Department of Pharmaceutics, College of Pharmacy, King Saud University, Riyadh 11451, Saudi Arabia; salshehri1@ksu.edu.sa; 6 Department of Pharmacy Practice, College of Pharmacy, AlMaarefa University, Ad Diriyah 13713, Saudi Arabia; mghoneim@mcst.edu.sa

**Keywords:** infectious disease, healthcare, artificial intelligence, disease treatment, computer methods

## Abstract

Artificial intelligence (AI) has been described as one of the extremely effective and promising scientific tools available to mankind. AI and its associated innovations are becoming more popular in industry and culture, and they are starting to show up in healthcare. Numerous facets of healthcare, as well as regulatory procedures within providers, payers, and pharmaceutical companies, may be transformed by these innovations. As a result, the purpose of this review is to identify the potential machine learning applications in the field of infectious diseases and the general healthcare system. The literature on this topic was extracted from various databases, such as Google, Google Scholar, Pubmed, Scopus, and Web of Science. The articles having important information were selected for this review. The most challenging task for AI in such healthcare sectors is to sustain its adoption in daily clinical practice, regardless of whether the programs are scalable enough to be useful. Based on the summarized data, it has been concluded that AI can assist healthcare staff in expanding their knowledge, allowing them to spend more time providing direct patient care and reducing weariness. Overall, we might conclude that the future of “conventional medicine” is closer than we realize, with patients seeing a computer first and subsequently a doctor.

## 1. Introduction

**Figure 1:**
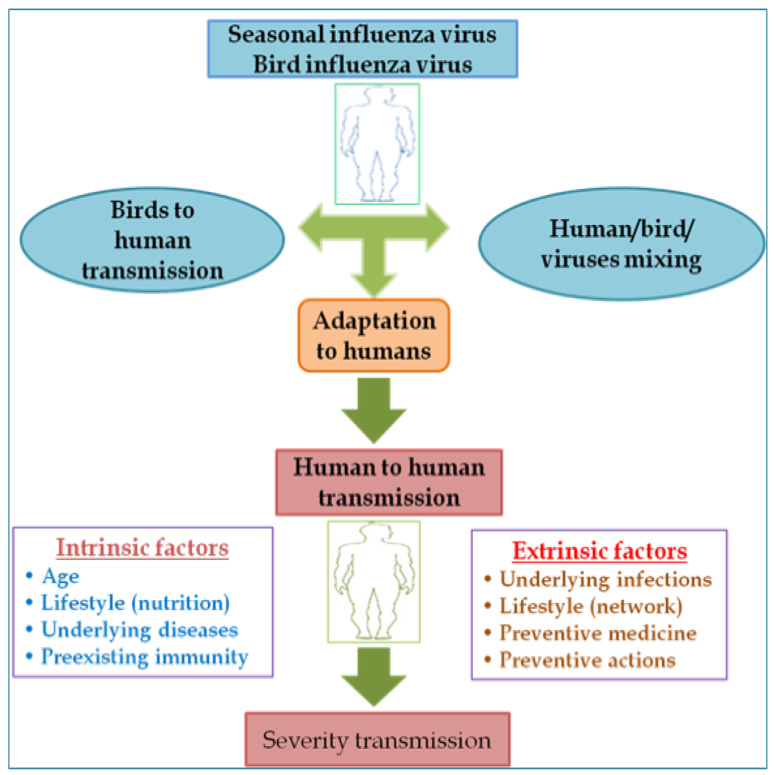
The mechanisms of influenza transmission and the factors influencing it.

Microorganisms that cause diseases, such as parasites, microbes, viruses, and fungi induce infectious illnesses and allow symptomatic or asymptomatic disorders to exist. Specific infectious conditions, for example, the human immunodeficiency virus (HIV), may be relatively asymptomatic but, if left untreated, may have catastrophic effects after a few years [1]. Disease-caused infections propagate in different ways depending on the microorganism. Infectious illnesses caused the greatest percentage of premature deaths and disabilities in the twentieth century. At the turn of the century, the Spanish flu made its appearance [2]. During the 1918–1919 pandemic, it is claimed that one-third of all people in the world (500 million people) were sick and had symptoms (Figure 1) [2]. It was one of the world’s most dangerous influenza pandemics in the history. At least 50 million people died as a result of the outbreak according to estimates [2]. Since then, nearly all outbreaks of influenza A were generated by mutant forms of the 1918 virus, and the pandemic’s effect was not limited to the first quarter of the twentieth century. 

**Figure 2:**
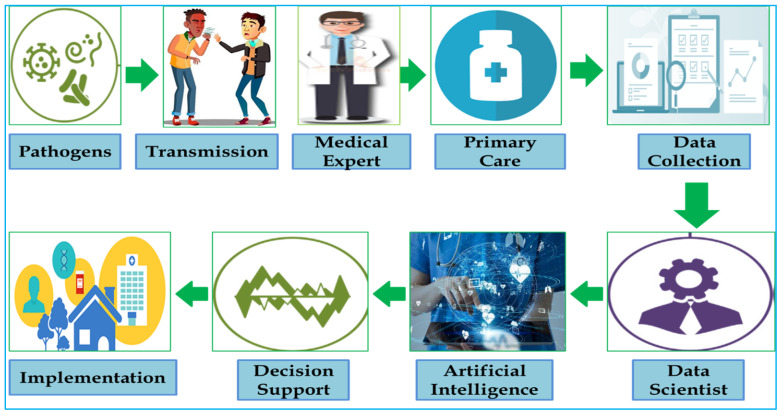
Infectious disease prevention principles must be followed. The series presents the main aspects of controlling infection and improving control by protective steps (vaccination and hygiene). The importance of the artificial intelligence (AI) environment in this effort cannot be overstated.

AI has been described as one of the extremely effective and successful scientific methods for humanity among the currently available tools [3]. Huge amounts of data are required to be washed, organized, and integrated into the input resource for AI. Recent studies have shown the importance of machine training for image recognition in situations where traditional technologies were unable to detect early symptoms of the disease [4]. This is especially true in case of the cancer [5], where AI will help with the diagnosis and treatment. This is also true in developed countries, where finances, healthcare costs, and other constraints hinder delivering adequate care. Focused on essential imaging and deep learning, a group of researchers recently demonstrated the feasibility of developing a reduced-price entrance of treatment for the diagnosis of lymphoma [6]. Numerous studies have proposed that Bayesian networks (BN) be used to describe dependencies in the statistics [7]. The BN is a charting framework of mutual multivariate probability distributions, which preserves conditional independence characteristics among different parameters [8]. The advancement of effective analytical methods is increasing in the period of biological systems and personalized drugs. A modern kind of information, known as recreational data, will become increasingly important in the healthcare field, which is the Internet of Things (IoT). The IoT is an increasing system of sensors and tools which gather the data and are used in our everyday lives. Wearable and smart systems are common examples of devices that produce constant data streams, which may be applied to have a deeper understanding of our way of life. It is claimed that over 7 billion linked devices are actually in use around the world, and using this technology will greatly expand the opportunities for improving our lives. Such datasets, as well as traditional wellness datasets, have been used to better understand infectious disorders, infection processes, treatment tolerance, spread, and vaccine layout (Figure 2) [7,8].

**Figure 3:**
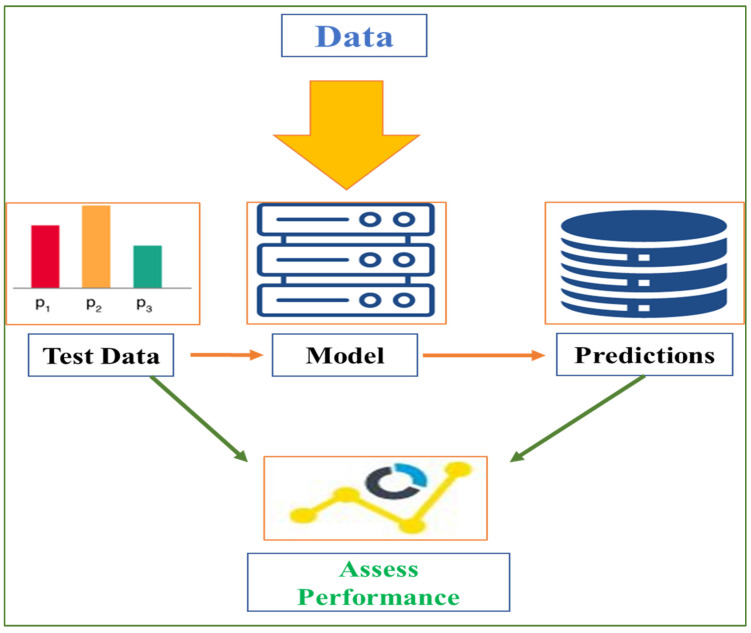
Methodology based performance assessment.

AI and its related advances are becoming more widely used in the business and society, and they are starting to show up in medical treatment. Many facets of healthcare, as well as the regulatory procedures within providers, payers, and pharmaceutical companies, may be transformed by these innovations. Various trials have also demonstrated that AI may perform as well as or better than humans in essential medical treatment activities such as the diagnosis of diseases. In terms of identifying cancerous cells and educating researchers on how to grow communities for costly clinical trials, algorithms are already outperforming radiologists. However, we believe it will be decades before AI replaces humans in a range of medical procedures for a variety of reasons. Although AI is poised to make a significant impact in healthcare, there are a few ethical problems to consider when putting these systems in place and making decisions about them. Accountability and openness in such systems’ decisions, the risk of team harm due to algorithmic prejudice and professional duties, and the integrity of therapists are only a few ethical concerns. As a result, it is critical to think about and analyze the possible benefits of high-quality healthcare systems with the most precise and cost-effective intelligence calculation at a very low cost when employing such programs. Furthermore, AI algorithms are capable of performing predictable computer analysis by filtering, modifying, and searching patterns on massive databases from several sources in order to make rapid and accurate conclusions. Therefore, in the present review, we discussed how AI can help with various aspects of healthcare, as well as some of the barriers to AI’s accelerated acceptance in healthcare, as described in (Figure 3) [6]. 

## 2. Types of AI Those Are Useful in Healthcare

AI is a group of technologies rather than a single technology. Many of these technologies are rapidly operating in the healthcare sector, but the specific procedures and functions they support may vary widely. Some of the most important AI healthcare technologies are described below.

### 2.1. Machine Learning and Deep Learning 

**Figure 4:**
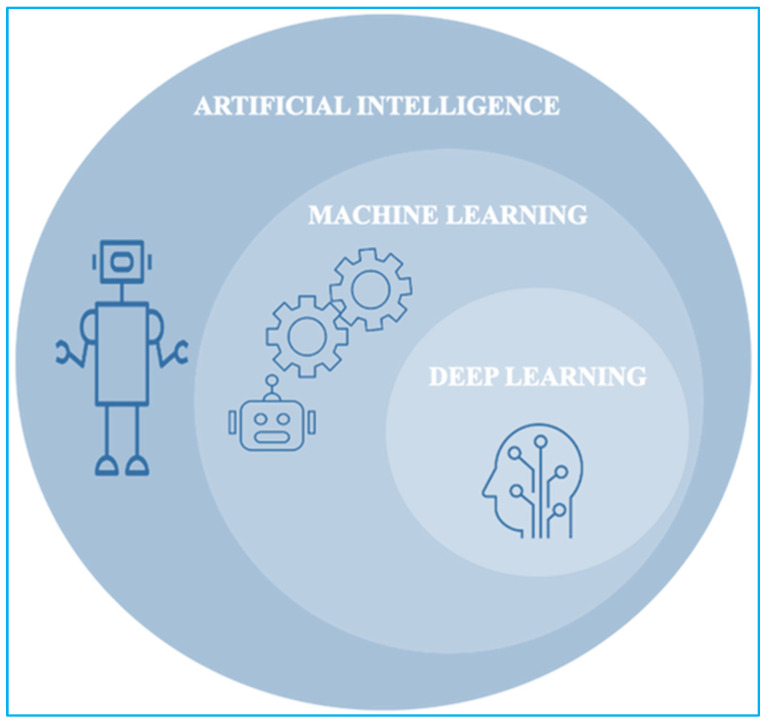
Process of machine and deep learning in AI.

Among in-depth learning and emotional networks, machine learning is a mathematical way of incorporating data models and teaching models to ‘learn’ by training them with the data (Figure 4) [9,10]. 

According to a 2018 Deloitte survey of 1100 US managers, whose organizations were already exploring AI, 63 percent of the companies surveyed were employing machine learning in their operations [9]. The most prevalent application of machine learning technology in healthcare is accurate medicine, which predicts treatment strategies that are likely to be beneficial for a patient based on their numerous features and treatment setting [10]. Most machine learning and accurate medicine applications require supervised reading, which is a training database with known variables, e.g., illness onset. The neural network is the most advanced kind of machine learning that has been accessible since the 1960s and has been well-established in the healthcare research for several decades [11]. It is used for isolated applications such as predicting whether a patient would become infected with a given disease. It searches for issues with inputs, outputs, and changeable weights or ‘features’ that connect outputs and inputs. It has been compared to how neurons process impulses, although the brain function parallel is not as strong. One of the most complicated techniques for machine learning is in-depth learning, which involves neural network models with several degrees of variability or variability that predicts the outcomes. The quick processing of contemporary image processing units and cloud architectures may reveal thousands of hidden elements in such models. A frequent in-depth research program in healthcare involves identifying probable malignant tumors in radiology imaging [12]. Radiomics, or the discovery of crucial clinical aspects in imaging data that is beyond the human eye’s purview, is increasingly used in in-depth studies [13]. In oncology-focused image analysis, both radiomics and in-depth learning are often used. Their combination appears to offer better diagnostic precision than the previous generation of image analysis technologies, known as computer-assisted detection (CAD). In-depth learning is also becoming more popular for speech recognition, and as a result, it is a type of natural language processing (NLP), which is covered further below. In contrast to the previous forms of mathematical analysis, each element in an in-depth reading model usually has little meaning for the observer. As a result, interpreting the model results may be too difficult or impossible. Recently, machine learning algorithms have been explored significantly in medical and public health diseases [14,15,16]. Machine learning algorithms are important in analyzing multiple and complex variable in clinical datasets [17,18,19]. The wide range of machine learning algorithms with different characters and design goals are available. Some advanced machine learning algorithms such as deep neural networks (DNN) and support vector machines (SVM) utilize complex nonlinear transformations in order achieve superior prediction accuracy [16,20,21]. However, it is not possible to figure out how these algorithms make predictions due to their complex nonlinear transformations. On the other hand, some machine learning algorithms such as decision trees (DT) and naïve Bayesian classifiers (NBC) follow highly interpretable decision processes to achieve the predictions [22,23,24,25]. DT and NBC offer inferior prediction accuracy compared to the DNN and SVM algorithms due to the absence of complex nonlinear transformations. All these algorithms are helpful in the prediction of accuracy in various infectious diseases. Several prediction models have also been developed for the identification of new COVID-19-infected patients [26,27,28]. 

### 2.2. Natural Language Processing

Since the 1950s, AI researchers have sought to understand human language. Speech recognition, text analysis, translation, and other language-related applications are all examples of NLP applications. There are two types of NLP: mathematical and semantic. Mathematical NLP is based on machine learning (particularly in-depth studies of neural networks) and has contributed to recent improvements in visual accuracy. You need to have a large ‘corpus’ or a language course in which you can learn. The most common use of NLP in healthcare involves the creation, understanding, and classification of clinical literature and published research. NLP systems can analyze randomized clinical notes on patients, prepare reports (for example, by radiology tests), record patient interactions, and conduct AI dialogue [11,29,30,31,32].

### 2.3. Robotic Process Automation

These technologies perform organized digital control tasks, such as those integrating information systems, as if human users are following a text or set of rules. It is less expensive, easier to configure, and more transparent in its behaviors than other types of AI. Robotic process automation (RPA) is a type of automation that uses computer programs that run on servers rather than robots. It employs workflow, business rules, and a combination of ‘presentation layer’ and information systems to function as a less intelligent programming user. They are used in healthcare to perform repetitive tasks such as prior authorization, updating patient records, and billing. When combined with other technologies such as image recognition, it can be used to extract the data from faxed images, for example, and be integrated into transaction systems [33]. These technologies are explained separately, but they are becoming increasingly intertwined: Robots are receiving sophisticated AI “brains,” while image recognition and RPA are merging. In the future, these technologies may become so intertwined that integrated solutions will become more or less feasible.

### 2.4. Explainable and Interpretable AI

Scientists often believe the terms explainability and interpretability to be interchangeable, but they have practical distinctions [34,35]. Although there is no formal mathematical definition for explainability or interpretability, several attempts to distinguish these two notions have been attempted [36,37,38]. The most common definition of explainability is the ability to communicate with humans in understandable terms [37]. The interpretability of a model’s outputs, on the other hand, is largely related to the intuition underlying the model’s outputs [39]. Explainable AI (XAI) aids in the communication of automated choices to affected patients in a clear and intelligible manner [34]. In the fields of healthcare and biomedical sciences, XAI is garnering more scientific interest [35]. The core logics and mechanics of a machine learning system are related to XAI. The more understandable model may lead to a deeper knowledge of human disorders. Humans may not be able to understand the internal logics or underlying mechanisms of an interpretable model [34,35]. Hence, when it comes to machine learning systems, interpretability does not imply explainability or vice versa. As a result, it has been suggested that interpretability alone is insufficient, and that the presence of explainability is also essential [37]. For a thorough grasp of XAI, a variety of models are provided [40,41,42,43,44]. Interpretable and interactive machine learning modeling approaches that engage both domain experts and machine learning experts simultaneously have also been utilized in healthcare systems [42,43]. 

### 2.5. Administrative Applications 

There are various management applications in healthcare. In this domain, the use of AI has less flexibility than inpatient care but can provide significant efficiency. This is required in healthcare because the average US nurse, for example, spends 25% of her time on the job on administrative duties [44]. RPA technology is most likely related to this goal. It has applications in a number of healthcare systems, including applicant processing, clinical recording, income cycle management, and medical records management [45,46]. In addition to the patient interaction, mental health and wellness, telehealth, and chatbots have been employed in additional healthcare settings. These NLP-based applications can help with tasks as easy as filling out a prescription or keeping track of a schedule. In a study of 500 US users of the top 5 health interviews, patients expressed concerns about disclosing private information, dealing with complex health circumstances, and misuse [47]. Machine learning is another AI technology associated with claims and payment management because it can be used to match possible data across the different websites. Insurance brokers are responsible for verifying the accuracy of the millions of the claims. Identifying, analyzing, and correcting incorrect coding problems and claims saves time, money, and effort for all stakeholders—health insurance, governments, and providers alike. Negative claims that slip through the gaps represent the enormous revenue opportunity that data comparisons and application evaluation tests can provide.

## 3. Diagnosis and Treatment Applications

MYCIN has been used to detect plasma infections since the 1970s when this was developed at Stanford [48]. The priority of AI has been on illness detection and therapy. Although, AI and many other early schemes built on rules have seen potential in terms of correctly diagnosing and illness treatment, they were never used in medical care. They were little different than human doctors and surgeons, and their processes and medical information processes were seriously organized. Numerous healthcare entities are having difficulty in implementing AI. While rule-based systems incorporated in electronic health record (EHR) systems are widely used, they lack the precision of more algorithmic frameworks based on machine learning [49]. More currently, IBM’s Watson has received a lot of press for their emphasis relating to precision medicine, especially tumor detection and medical care. Watson uses a mixture of AI and NLP. Nonetheless, the consumers’ support for the use of engineering has waned when they realize how difficult it is to educate Watson on how to deal with specific forms of cancer, as well as how difficult it is to integrate Watson into the treatment procedures and programs. Watson is a set of “cognitive resources” offered via the application programming interfaces (APIs), including speech and language vision, as well as data analysis and machine learning algorithms [50]. Based on the rules, the medical choice support mechanisms are complex to manage when medical science evolves, and they are frequently incapable of handling the avalanche of evidence and information resulting from genetic, proteomic, biochemical, and other ‘omics-based’ approaches to the treatment. Most of the certain conclusions are centered on the radiological image processing [51], while some use other kinds of photographs, such as retinal scanning [52] or precision medicine based on genomic data [53]. These kinds of results that are founded on computer-focused statistical techniques for study are being announced in a phase of proof- and probability-based science, which is widely viewed as optimistic but poses numerous ethical issues in medicine and relationships between the patients and doctors [54]. The companies in the technology sector and entrepreneurs are now concentrating their energies on the same issues. For example, Google is collaborating with healthcare providers channels to develop big-data predictive models that would alert physicians to elevated conditions such as sepsis and heart disease [55]. Image-understanding algorithms based on Google, Enlitic, and a host of other firms are working on AI. Jvion developed a “clinical progress machine” that recognizes the patients who are the most in danger and those that are most expected to react to treatment protocols. Each of these may help physicians make better decisions when it comes to determining the right diagnosis and care for their patients [54].

## 4. Applications for Patient Involvement and Adherence

Patient involvement and compliance have long been considered the “last mile” barrier in the healthcare industry, the last line of defense between poor and good health outcomes. The better the outcomes—utilization, cost, and member experience—the more patients take an active role in their health and care. In a study of more than 300 physician healthcare executives and legislators, more than 70% of those who responded claimed that fewer than half of their patients were actively interested, and 42% said that less than a quarter of their customers were deeply connected [56]. Can AI-based abilities be successful in enhancing and contextualizing treatment if better patient engagement results in improved healthcare results? Machine learning and workflow engines are increasingly being used to guide complex interventions across the healthcare spectrum [57]. Messaging warnings and associated, tailored material that motivates behavior at critical moments is an attractive field of research.

## 5. Implications for the Healthcare Workforce

The concern about how AI might lead to process advancement and major job losses has received a lot of press, as per a Deloitte–Oxford Martin Institute collaboration [58]. AI might generate 35 percent of UK jobs in the next 10 to 20 years. Multiple surveys have found that, while certain tasks can be automated, several other factors, such as the cost of robotics advancements, labor sector advancement and cost, automation has several benefits, and approval on a national and social level may help to prevent job losses [59]. Automation has several benefits, beyond simple labor substitution, and approval on a national and social level may help to prevent job losses. Jobs that count losses can be restricted to 5% or minute due to these causes. Rather than actual patient touch, the healthcare jobs that include dealing with electronic signatures tend to be most probably to be automated, for example, radiography and pathology [60]. There is also a chance that new positions will be developed to support and improve AI technology. However, AI systems are unlikely to significantly decrease the rates of clinical diagnosis and care throughout the period if individual jobs remain stable or increase. On the other hand, bias is one of the major issues of AI systems, which cannot be ignored. The details of bias with respect to AI systems are described below.

### 5.1. Bias 

This is not a new problem, but a “bias as old as a human civilization”; it is human nature that most members of the ruling party ignore the experience of other parties. However, AI-based decision-making has the potential to magnify the existing biases and transform new categories and conditions, which may lead to new types of bias. These ever-increasing concerns have led to the re-evaluation of AI-based programs to implement new approaches that address the impartiality of their decisions. The latest technologies for bias in AI-based decision-making systems, as well as open challenges and guidelines for AI solutions for the public good, are discussed. Bias is divided into three broad categories.

#### 5.1.1. Understanding Bias 

Methods that can be adjusted and explicitly described to aid in understanding how bias is formed in society and to integrate into our sociotechnical systems manifest themselves in data utilizing AI algorithms and can be modeled and formally defined.

##### Mitigating Bias 

Pre-processing, processed, and post-processing strategies addressed bias in the various stages of AI decision making, with pre-processing, processed, and post-processing methods focusing on data entry, learning algorithms, and model results, respectively.

##### Accounting for Bias 

Methods that introduce bias either in the present, as a result of data collecting bias, or in the past, as a result of interpreting AI judgments in human terms. We know that bias and prejudice are not limited to AI and that technology can be used (consciously or unconsciously) to reflect, enhance, or distort the real-world perspective. As a result, it is naive to believe that technological fixes will suffice, because the core causes of these issues are not limited to technology. To ensure the long-term well-being of all parties, more technological solutions are needed, including socially acceptable definitions of fairness and reasonable interventions. Fairness is critical machine learning in AI [61]. It is well known that algorithms are not fair in minority sub-populations [62]. Fairness has important role in bias. The fairness in multiaccurcay boost could reduce the chances of different kind of bias [62]. Because bias and discrimination are multifaceted and flexible, these challenges require multidisciplinary perspectives and ongoing dialogue with the society [63]. However, as AI technology enters our lives, it is important for technology creators to recognize bias and prejudice and ensure responsible technology use, keeping in mind that technology alone is not the solution to all forms of bias and AI problems [64].

### 5.2. Various Implications for the Healthcare Workforce

In the healthcare, the circular data processing is used to produce sound communicable decisions. The increasing growth in clinical data has added to the stress of healthcare employees’ jobs, limiting their capacity to offer high-quality and efficient care. Healthcare organizations should reconsider their tactics to ensure that staff are completely satisfied and supported in their work. The use of AI has the potential to improve operator performance. The use of AI in healthcare is not new, but it has made the great strides in the field in recent years. This has been made possible in part by substantial advancements in big data analysis, which have been supported by the increasing access to healthcare data. When used in conjunction with proper analytical methodologies, such as machine learning tools, AI has the potential to alter many aspects of the healthcare.

### 5.3. AI to the Rescue

AI may alter the function of healthcare providers and, as a result, the interaction between them and their patients. On the one hand, as automation grows in power, there is fear about the future, but there is also concern that increased technological output would render some healthcare services obsolete [65]. While much remains unknown about how AI will be implemented, there are signs that AI has the ability to improve provider performance in terms of providing effective, efficient, and high-quality care.

### 5.4. Productivity

Administrative duties, data extraction from health records, treatment plan design, and consulting are just a few of the applications where AI is applied. Some time-consuming repetitive processes can be performed quickly and effectively using AI. This allows healthcare practitioners to dedicate more time to the treatments that are tailored to the clinical conditions and demands of their patients. Furthermore, AI enables healthcare providers to oversee the care of huge groups of the patients. The adoption of AI-enabled tools in nursing has been shown to enhance the productivity by 30–50 percent [66]. A strong technique to meet the basic goal of healthcare has been suggested: combining AI and human intellect, or ‘extended intelligence’ [67].

### 5.5. Workload

Work stress, which impacts the quality of care and patient outcomes, accounts for a large portion of the workload [68,69]. Previous research has revealed that administrative responsibilities have a significant impact on worker turnover and time constraints [68]. In ambulatory settings, for example, physicians spend 49 percent of their time on electronic and desktop health data, whereas only 33 percent of their time is spent on direct clinical contact with the patients and staffs [70,71]. AI has the ability to dramatically reduce the administrative load by automatically filling in structured data fields from open clinical notes, accessing essential data from previous clinical records, and collecting recorded patient encounters. According to a recent study [72], voice-to-text writing will save doctors 17 percent of their time and registered nurses 51 percent of their time. Amazon is working on a new machine learning solution that will extract useful information from unstructured EHR data and unstructured clinical notes. Amazon’s medical comprehension enables the extraction of essential clinical terminology relating to patient diagnosis, drugs, symptoms, treatment, and other interactions with the healthcare system from unstructured EHR data [73].

### 5.6. Performance

AI systems have the potential to improve the diagnostic and treatment decisions, while reducing medical errors. In the fields of medical imaging and diagnostics, AI has made great progress. In-depth learning techniques aid in the prevention of diagnostic errors and the improvement of test results. For example, AI has been shown to improve the clinical imaging investigations in the detection of cancer and diabetic retinopathy [74,75]. Many healthcare providers are incorporating AI into their everyday routines in order to obtain knowledge on an increasing amount of clinical data and therefore reduce patient risk. AI can also be utilized to update clinical records automatically, retrieve quality reporting data, and insert diagnostic codes [76]. Furthermore, technology companies such as Google (DeepMind), IBM (Watson), and others are investigating the possibility of AI-enabled surgical robots that use machine learning skills. The use of AI-enabled robots is intended to increase accuracy, reduce harm, and accelerate therapeutic recovery.

### 5.7. Teamwork

The current status of healthcare necessitates cooperation and collaboration between healthcare providers. To promote collaborative decision making, coordinated activities, and progress tracking, excellent communication is required. AI may combine the data from a variety of formal and informal sources to provide the integrated, quick, and consistent access to the patient data across the numerous settings and instructions. Chatbots have been used to arrange and coordinate the therapy sessions, provide reminders, and educate the physicians on the patient’s condition based on the symptoms in some cases.

### 5.8. Newer Challenges

AI has the potential to significantly improve the quality and efficiency of healthcare, resulting in increased productivity, provider satisfaction, and user experience, as well as better outcomes. Policymakers, industry, healthcare providers, and patients must all face new obstacles in order to fully grasp AI’s potential.

### 5.9. Professional Liability

Traditionally, clinical decision making has been the purview of the licensed healthcare specialists. Because AI is frequently utilized to aid with clinical operations, AI decision support systems may have an impact on the professional obligations of healthcare practitioners in each patient. Given AI’s ability to make incorrect conclusions, the legal obligation of AI-assisted decisions is frequently misinterpreted. This is complicated further by the fact that developing relevant legal concepts and guidelines takes longer than developing technological skills. Another fear is that AI may deter healthcare providers, preventing them from double-checking results and challenging inaccuracies [77].

### 5.10. Labour Market Implications

The skills and competence required by healthcare providers are anticipated to alter as a result of the introduction of various new technologies. In some cases, AI may be able to perform tasks that humans previously performed. Furthermore, as AI progresses in healthcare, new skill sets, such as informatics, may become more in demand. To satisfy the needs of the labor market, education, and training programs, it will need to be adjusted. There are also concerns that AI systems will be used to justify the hiring of low-skilled personnel. If technology fails and staff are unable to spot the mistakes or accomplish needed duties without the assistance of computers, this might be troublesome [78,79].

### 5.11. Provider Competencies

The skills and competence required by the healthcare providers are anticipated to alter as a result of the introduction of various new technologies. In some cases, AI may be able to perform tasks that humans previously performed. Furthermore, as AI progresses in healthcare, new skill sets, such as informatics, may become more in demand. To satisfy the needs of the labor market, education, and training programs will need to be adjusted. There are also concerns that AI systems will be used to justify the hiring of low-skilled personnel. 

## 6. Ethical Implications

Finally, AI’s application in healthcare creates a slew of legal issues. Humans used to make almost all healthcare decisions, so having smart technologies produce or assist with them raises issues of accountability, transparency, consensus, and secrecy [80]. With today’s technology, the most difficult problem to tackle is transparency. Most AI algorithms, in particular machine training algorithms utilized during image manipulation, are nearly inaccessible to understand or interpret [81]. If a person told that a picture contributed to the detection of a tumor, she or he would almost certainly want to know why. Extreme thinking algorithms, as well as doctors with a basic understanding of how they work, may be unable to offer an interpretation. Inpatient treatment and diagnosis will almost certainly be messed up by AI software, and holding them accountable could be tough. Patients are more likely to receive care records from AI systems than from a knowledgeable physician. Machine learning models in healthcare may be prone to algorithmic bias, such as predicting a higher risk of disease based on sex or ethnicity when such elements are not causal factors [80,81].

Computer systems are an important branch of ethics that began to emerge in the late 1950s and early 1960s. It arose as a result of the introduction of computers and the moral implications that resulted. Computer ethics is about the effects of ethical behavior on the existence and use of computers. In healthcare, AI has many behavioral effects. The first behavioral problem is that of AI’s ethical responsibility. A moral obligation is an obligation to accept responsibility for one’s actions. Some may argue that they have no moral obligation because AI is sensitive. It is important to note, however, that AI may be morally responsible. For example, the computer program used in medical examinations is not emotional, but it has the moral obligation to do so. The second moral error is the responsibility of the AI developer. It is responsible for ensuring that AI is able to meet people’s needs. The third difficulty of behavior is the responsibility that comes with using AI. It is our responsibility to ensure that AI is not used for unethical purposes. Responsibility related to people affected by AI is the fourth behavioral problem. AI is responsible for ensuring that it does not have a negative impact on a particular group of people or society. The fifth meaning of ethics is the responsibility that comes with using AI. It is our responsibility to ensure that AI is not used in ways that infringe on the rights of others. The responsibility associated with the ethics used to guide AI design is the sixth ethical riddle. These principles are used to help AI developers ensure that AI works. 

### 6.1. Six Principles to Ensure that AI Serves the Public Interest in All Countries

The WHO provides the following principles as the basis for AI control and governance in order to limit the risks and maximize the potential for the use of AI in healthcare [82]:

#### 6.1.1. Protecting Human Autonomy

This means that people should govern healthcare systems and medical decisions; privacy and confidentiality must be safeguarded, and patients must give informed consent utilizing proper legal frameworks for data protection. Information sharing agreements could be utilized to provide the institutions access to the health information of the patients [83]. It is known that some public–private partnerships for implementing machine learning have resulted in the poor protection of privacy [84]. Therefore, the privacy aspects of healthcare in AI and machine learning must be safeguarded [83,84]. 

#### 6.1.2. Promoting Human Well-Being and Safety, as well as the Public Interest

Regulatory standards for the safety, accuracy, and efficacy of well-defined application cases or indicators must be met by AI technology designers. Measures to increase the quality of AI use and control practice performance should be in place [83].

#### 6.1.3. Importance of Transparency, Explainability, and Intelligibility

To demonstrate this, sufficient material must be published or written prior to the invention or deployment of AI technology. Such data should be readily available to allow for real public participation and debate regarding how technology is built and how it should or should not be utilized [84].

#### 6.1.4. Fostering Responsibility and Accountability

Although AI technology is capable of performing certain functions, it is the responsibility of participants to ensure that it is used under the right conditions and properly trained people. Individuals and groups affected by algorithm-based decisions should have access to effective question-and-answer methods [82].

#### 6.1.5. Ensuring Inclusiveness and Equity

Involvement requires that AI health is designed to promote the use and access to equity as broadly as possible, regardless of age, gender, sex, income, race, ethnicity, sexual orientation, ability, or other aspects protected by human rights [84].

#### 6.1.6. Promoting AI that Is both Responsive and Sustainable

Designers, engineers, and users should evaluate AI systems on a regular basis and in public to see if it responds adequately and effectively to expectations and requirements. AI algorithms should also be designed to have the least amount of environmental impact and to use as little energy as feasible. Governments and businesses should plan for potential workplace disruptions, such as the training of healthcare personnel to adapt to AI systems and the potential loss of jobs as a result of automated systems. These principles will guide future WHO efforts to guarantee that AI’s full promise for healthcare and public health is realized [82].

## 7. AI in Disaster Management

In today’s world, AI has exploded in popularity. The use of AI as a tool could help to lower the danger of death, environmental damage, and societal impact, as well as respond to disasters more intelligently [85]. The function of AI in disaster management is critical for predicting scenarios and determining disaster solutions. By minimizing the risk of human life during disasters, AI fosters technological growth and stimulates development [86]. It has been suggested that AI be used in order to offer reliable results based on the algorithms stored in the AI technology database. It is critical to acquire data from previous catastrophes in order to conduct an analysis and design efficient disaster mitigation strategies [85,86,87]. Various AI difficulties have been identified, as a machine cannot possess all of the characteristics of a human. The expenses, protection of human life, environmental protection, and incorrect data are among the challenges. The development of AI comes at a high cost [85,86]. Advanced technologies, strong companies, professional expertise, and extensive testing are all required. Testing vulnerabilities for disaster management takes time and money in the creation of drones and robotics [85]. Both animal and human life are affected by disasters. As a result, programming in AI technologies must be precise in order to predict any impending danger. At the same time, it is critical to preserve lives and prevent deaths during a disaster [85,86]. AI is designed to maintain the ecosystem, and protecting the entire environment on such a large scale during a disaster is tough. Before adopting any corrective efforts, the disaster-affected area must be examined, and infrastructure, society, and the environment will all suffer losses. The aim is to conserve and remodel everything that has been impacted following a calamity, which necessitates advanced AI skills. Real-time data can save lives, but it is difficult to obtain such data, and any erroneous information can be fatal [85]. Erroneous information has negative implications for catastrophe preparedness and response. The actual crowd is represented by the data collected from multiple sources, which includes both purposeful and unintentional misleading data [85,86].

## 8. Conclusions

We believe that AI will play a significant role in the healthcare industry. Precision medicine, which is widely known for much improvement in healthcare, is fueled by this capability. Though early attempts at diagnosis and therapy guidance proved challenging, we expect that AI can now master the domain as well. Thanks to significant improvements in AI for imaging science, many radiology and pathology images are projected to be evaluated by a device at some point. Voice and text analytics are increasingly used for tasks such as patient communication and diagnostic report collection, and this pattern is expected to continue. The most challenging challenge for AI in such health domains is maintaining its acceptance in ordinary clinical practice, rather than whether the technologies are competent enough to be effective. AI programs should be licensed by governing bodies, capable of EHR systems, standardized to the point that identical devices function in the same way, trained by physicians, financed by either public or commercial payers, and modified in the long run in the sector for universal acceptance to occur. These obstacles will be solved in the end, but they will require even more than that for the maturation of the technology itself. As a result, within the next five years, we expect to see minimal AI application in clinical practice, with more widespread use by the next decade. It can increase the productivity and efficiency of care delivery and allow healthcare systems to provide more and better care for more people. Compared to previously reported articles on AI, this review focused on the applications of AI in healthcare systems especially in the diagnosis and treatment of infectious diseases (Table 1). Based on the summarized data, it has been inferred that the AI can help in improving the knowledge of healthcare workers, enabling them to spend more time in direct patient care and reduce fatigue. Overall, we might conclude that the future of ‘traditional medicine’ may be closer than we think, with patients first seeing a computer and then a doctor.

## 9. The Future of AI in Healthcare

We believe that AI will play an important role in future healthcare delivery. A critical talent in the development of precise medicine, which is universally recognized as a much-needed advance in healthcare. Although early attempts to make diagnostic and therapeutic advice proved tough, we believe AI will finally grasp the subject. Given the rapid development of imaging techniques, it appears that most radiology and pathology images will be scanned by machines at some point. Speech and text recognition are currently in use for things such as patient communication and clinical photography, and they will continue to grow in popularity. The most difficult challenge for AI in various healthcare settings is assuring its availability in day-to-day clinic operations, not whether the technology will be useful. To achieve widespread acquisition, AI programs must be approved by regulators, integrated with EHR systems, standardized until the same products do the same, trained by physicians, paid for by public or private organizations, and updated in the field over time. These obstacles will be solved eventually, but it will take longer for the technology to evolve. As a result, we anticipate modest AI applications in clinical practice during the next five years, followed by widespread adoption over the next decade. It is also evident that AI algorithms will not, on a big scale, replace human doctors but will instead intensify their efforts to care for patients. Human physicians may eventually switch to careers that require specialized human skills such as empathy, persuasion, and the integration of large images. Those healthcare providers who refuse to cooperate with AI may be the only ones who lose their jobs over the time.

## Figures and Tables

**Table 1:** Application of different kinds of artificial intelligence (AI) in the prevention and diagnosis of different diseases.

S.N.	Type of AI	Application	Reference
1	AI	Clinical oncology	[5]
2	Machine learning	Lymphoma	[6]
3	Machine learning	Myeloid leukemia	[10]
4	Deep learning	Cancer	[13]
5	AI	COVID-19	[14]
6	Machine learning	Dengue	[15]
7	Machine learning	Cardiovascular diseases	[16]
8	Deep learning	Pulmonary infection	[19]
9	Deep learning	COVID-19	[27]
10	Machine learning	Venous thromboembolism	[40]
11	Machine learning	Neovascular macular degeneration	[53]

## References

[1] World Health Organization (2021). Infection Prevention and Control. https://www.who.int/health-topics/infection-prevention-and-control#tab=tab_1.

[2] Taubenberger J.K., Morens D.M. (2006). 1918 influenza: The mother of all pandemics. Emerg. Infect. Dis..

[3] Silver D., Schrittwieser J., Simonyan K., Antonoglu I., Huang A., Guez A., Hubert T., Baker L., Lai M., Bolton A. (2017). Mastering the game of Go without human knowledge. Nature.

[4] Chen J.H., Asch S.M. (2017). Machine learning and prediction in medicine—Beyond the peak of inflated expectations. N. Engl. J. Med..

[5] Boon I.S., Yong T.P.T.A., Boon C.S. (2018). Assessing the role of artificial intelligence (AI) in clinical oncology: Utility of machine learning in radiotherapy target volume delineation. Medicines.

[6] Im H., Pathania D., McFarland P.J., Sohani A.R., Degani I., Allen M., Coble B., Kilcoyne A., Hong S., Rohrer L. (2018). Design and clinical validation of a point-of-care device for the diagnosis of lymphoma via contrast-enhanced microholography and machine learning. Nat. Biomed. Eng..

[7] Xu J., Wickramarathne T.L., Chawla N.V. (2016). Representing higher-order dependencies in networks. Sci. Adv..

[8] Belle A., Kon M.A., Najarian K. (2013). Biomedical informatics for computer-aided decision support systems: A survey. Sci. World J..

[9] (2018). Deloitte Insights State of AI in the Enterprise Deloitte. https://www2.deloitte.com/content/dam/insights/us/articles/4780_State-of-AI-in-the-enterprise/DI_State-of-AI-in-the-enterprise-2nd-ed.pdf.

[10] Lee S.I., Celik S., Logsdon B.A., Lundberg S.M., Martins T.J., Oehler V.G., Estey E.H., Miller C.P., Chien S., Dai J. (2018). A machine learning approach to integrate big data for precision medicine in acute myeloid leukemia. Nat. Commun..

[11] Sordo M. (2002). Introduction to Neural Networks in Healthcare. Open Clin..

[12] Fakoor R., Ladhak F., Nazi A., Huber M. Using deep learning to enhance cancer diagnosis and classification. A conference presentation. Proceedings of the 30th International Conference on Machine Learning.

[13] Vial A., Stirling D., Field M., Ros M., Ritz C., Carolan M., Holloway L., Miller A.A. (2018). The role of deep learning and radiomic feature extraction in cancer-specific predictive modelling: A review. Transl. Cancer Res..

[14] Pourhomayoun M., Shakibi M. (2021). Predicting mortality risk in patients with COVID-19 using artificial intelligence to help medical decision-making. Smart Health.

[15] Ho T.S., Weng T.-C., Wang J.-D., Han H.-C., Cheng H.-C., Yang C.-C., Yu C.-H., Liu Y.-J., Hu C.H., Huang C.-Y. (2020). Comparing machine learning with case-control models to identify confirmed dengue cases. PLoS Negl. Trop. Dis..

[16] Weng S.F., Reps J., Kai J., Garibaldi J.M., Qureshi N. (2017). Can machine-learning improve cardiovascular risk prediction using routine clinical data?. PLoS ONE.

[17] Chiu H.-Y.R., Hwang C.-K., Chen S.-Y., Shih F.-Y., Han H.-C., King C.-C., Gilbert J.R., Fang C.-C., Oyang Y.-J. (2022). Machine learning for emerging infectious disease field responses. Sci. Rep..

[18] Alfred R., Obit J.H. (2021). The role of machine learning methods in limiting the speed of deadly diseases: A systematic review. Heliyon.

[19] Zhang Y.-H., Hu X.-F., Ma J.-C., Wang X.-Q., Luo H.-R., Wu Z.-F., Zhang S., Shi D.-J., Yu Y.-Z., Qiu X.-M. (2021). Clinical applicable AI system based on deep learning algorithm for differentiation of pulmonary infectious disease. Front. Med..

[20] James G., Witten D., Hastie T., Tibshirani R. (2013). An Introduction to Statistical Learning.

[21] Cortes C., Vapnik V. (1995). Support-vector networks. Mach. Learn..

[22] Cho S., Hong H., Ha B.-C. (2010). A hybrid approach based on the combination of variable selection using decision trees and casebased reasoning using the Mahalanobis distance: For bankruptcy prediction. Expert Syst. Appl..

[23] Therneau T.M., Atkinson B., Ripley M.B. (2010). The Rpart Package.

[24] Maimon O.Z., Rokach L. (2014). Data Mining with Decision Trees: Theory and Applications.

[25] Sa-Ngamuang C., Haddawy P., Luvira V., Piyaphanee W., Iamsirithaworn S., Lawpoolsri S. (2018). Accuracy of dengue clinical diagnosis with and without NS1 antigen rapid test: Comparison between human and Bayesian network model decision. PLoS Negl. Trop. Dis..

[26] Kim H.-J., Han D., Kim J.-H., Kim D., Ha B., Seong W., Lee Y.-K., Lim D., Hong S.O., Park M.-J. (2020). An easy-to-use machine learning model to predict the prognosis of patients with COVID-19: Retrospective cohort study. J. Med. Internet Res..

[27] Liang W., Yao J., Chen A., Lv Q., Zanin M., Liu J., Wong S., Li Y., Lu J., Liang H. (2021). Early triage of critically ill COVID-19 patients using deep learning. Nat. Commun..

[28] Wu G., Yang P., Xie Y., Woodruff H.C., Rao X., Guiot J., Frix A.-N., Louis R., Moutschen M., Li J. (2020). Development of a clinical decision support system for severity risk prediction and triage of COVID-19 patients at hospital admission: An international multicentre study. Eur. Respir. J..

[29] Esteva A., Robicquet A., Ramsundar B., Kuleshov V., DePristo M., Chou K., Cui C., Corrado G., Thrun S., Dean J. (2019). A guide to deep learning in healthcare. Nat. Med..

[30] Bresnick J. (2020). “What is the Role of Natural Language Processing in Healthcare?” Health IT Analytics. https://healthitanalytics.com/features/what-is-the-role-of-natural-language-processing-in-healthcare.

[31] Juhn Y., Liu H. (2020). Artificial intelligence approaches using natural language processing to advance EHR-based clinical research. J. Allerg. Clin. Immunol..

[32] Rangasamy S., Nadenichek R., Rayasam M., Sozdatelev A. (2018). Natural Language Processing in Healthcare. McKinsey & Company. https://www.mckinsey.com/industries/healthcare-systems-and-services/our-insights.

[33] Hussain A., Malik A., Halim M.U., Ali A.M. (2014). The use of robotics in surgery: A review. Int. J. Clin. Pract..

[34] Lotsch J., Kringel D., Ultsch A. (2022). Explainable artificial intelligence (XAI) in biomedicine: Making AI decisions trustworthy for physicians and patients. Biomedinformatics.

[35] Linardatos P., Papastefanopoulos V., Kotasiantis S. (2021). Explainable AI: A review of machine learning interpretability methods. Entropy.

[36] Lipton Z.C. (2018). The mythos of model interpretability. Queue.

[37] Doshi-Velez F., Kim B. (2017). Towards a rigorous science of interpretable machine learning. arXiv.

[38] Gilpin L.H., Bau D., Yuan B.Z., Bajwa A., Specter M., Kagal L. Explaining explanations: An overview of interpretability of machine learning. Proceedings of the 2018 IEEE 5th International Conference on Data Science and Advanced Analytics (DSAA).

[39] Adadi A., Berrada M. (2018). Peeking inside the black-box: A survey on explainable artificial intelligence (XAI). IEEE Access.

[40] Datta A., Matlock M.K., Dang N.L., Moulin T., Woeltje K.F., Yanik E.L., Swamidass S.J. (2021). Black box’ to ‘conversational’ machine learning: Ondansetron reduces risk of hospital-acquired venous thromboembolism. IEEE J. Biomed. Health Informat..

[41] Datta A., Flynn N.R., Barnette D.A., Woeltje K.F., Miller J.P., Swamidass S.J. (2021). Machine learning liver-injuring drug interactions with non-steroidal anti-inflammatory drugs (NSAIDs) from a retrospective electronic health record (EHR) cohort. PLoS Comput. Biol..

[42] Shamshirband S., Fathi M., Dehzangi A., Chronopoulos A.T., Alinejad-Rokny H. (2021). A review on deep learning approaches in healthcare systems: Taxonomies, challenges, and open issues. J. Biomed. Infomat..

[43] Naser M.Z. (2021). An engineer’s guide to eXplainable artificial intelligence and interpretable machine learning: Navigating causality, forced goodness, and the false perception of inference. Autom. Const..

[44] Dikshit A., Pradhan B. (2021). Interpretable and explainable AI (XAI) model for spatial drought prediction. Sci. Total Environ..

[45] Berg S. (2018). Nudge Theory Explored to Boost Medication Adherence.

[46] Commins J. (2010). Nurses Say Distractions Cut Bedside Time by 25%. Health Leaders. www.healthleadersmedia.com/nursing/nurses-say-distractions-cut-bedside-time-25.

[47] Utermohlen K. (2018). Four robotic Process Automation (RPA) Applications in the Healthcare Industry. Medium. https://medium.com/@karl.utermohlen/4-robotic-process-automation-rpa-applications-inthe-healthcare-industry-4d449b24b613.

[48] Buchanan B.G., Shortliffe E.H. (1984). Rule-Based Expert Systems: The MYCIN Experiments of the Stanford Heuristic Programming Project.

[49] Davenport T.H. (2018). The AI Advantage Cambridge.

[50] Ross C., Swetlitz I. (2017). IBM Pitched Its Watson Supercomputer as a Revolution in Cancer Care. It’s Nowhere Close Stat. www.statnews.com/2017/09/05/watson-ibm-cancer.

[51] Right Care Shared Decision Making Programme, Capita (2012). Measuring Shared Decision Making: A Review of Research Evidence. NHS. www.england.nhs.uk/wp-content/uploads/2013/08/7sdm-report.pdf.

[52] Loria K. (2018). Putting the AI in Radiology. Radiol. Today.

[53] Schmidt-Erfurth U., Bogunovic H., Sadeghipour A., Schlegl T., Langs G., Gerendas B.S., Osborne A., Waldstein S.M. (2018). Machine learning to analyze the prognostic value of current imaging biomarkers in neovascular age-related macular degeneration. Opthamol. Retina.

[54] Aronson S., Rehm H. (2015). Building the foundation for genomic-based precision medicine. Nature.

[55] Rysavy M. (2013). Evidence-based medicine: A science of uncertainty and an art of probability. Virtual Mentor.

[56] Davenport T.H., Hongsermeier T., Mc Cord K.A. (2018). Using AI to Improve Electronic Health Records Harvard Business Review. https://hbr.org/2018/12/using-ai-to-improve-electronic-health-records.

[57] Volpp K.G., Mohta N.S. (2016). Patient Engagement Survey: Improved Engagement Leads to Better Outcomes, but Better Tools Are Needed. Insights Report. NEJM Catalyst. https://catalyst.nejm.org/doi/full/10.1056/CAT.16.0842#:~:text=in%20the%20world.-,Patient%20Engagement%20Survey%3A%20Improved%20Engagement%20Leads%20to%20Better%20Outcomes%2C%20but,appear%20to%20be%20the%20norm..

[58] User Testing (2019). Healthcare Chatbot Apps Are on the Rise but the Overall Customer Experience (cx) Falls Short According to a UserTesting Report.

[59] (2015). Deloitte from Brawn to Brains: The Impact of Technology on Jobs in the UK. Deloitte. www2.deloitte.com/content/dam/Deloitte/uk/Documents/Growth/deloitte-uk-insights-from-brawns-to-brain.pdf.

[60] McKinsey Global Institute (2017). A Future that Works: Automation, Employment, and Productivity McKinsey Global Institute. www.mckinsey.com/~/media/mckinsey/featured%20insights/Digital%20Disruption/Harnessing%20automation%20for%20a%20future%20that%20works/MGI-A-future-that-works-Executive-summary.ashx.

[61] Obermeyer Z., Powers B., Vogeli C., Mullainathan S. (2019). Dissecting racial bias in an algorithm used to manage the health of populations. Science.

[62] Kim M.P., Ghorbani A., Zou J. (2019). Multiaccuracy: Black-box-post-processing for fairness in classification. AIES.

[63] Atzmueller M. (2018). Declarative aspects in explicative data mining for computational sense making. Declarative Programming and Knowledge Management.

[64] Guidotti R., Monreale A., Ruggieri S., Turini F., Giannotti F., Pedreschi D. (2019). A survey of methods for explaining black box models. ACM Comput. Surv..

[65] Kletzer L.G. The question with AI isn’t whether we’ll lose our jobs—It’s how much we’ll get paid. https://hbr.org/2018/01/the-question-with-ai-isnt-whether-well-lose-our-jobs-its-how-much-well-get-paid.

[66] Robert N. (2019). How artificial intelligence is changing nursing. Nurs. Manag..

[67] Davenport T.H., Glover W.J. (2018). Artificial intelligence and the augmentation of health care decision-making. N. Engl. J. Med. Catalyst..

[68] Marine A., Ruotsalainen J.H., Serra C., Verbeek J.H. (2006). Preventing occupational stress in healthcare workers. Cochrane Database Syst. Rev..

[69] Nieuwenhuijsen K., Bruinvels D., Frings-Dresen M. (2010). Psychosocial work environment and stress related disorders, a systematic review. Occup. Med..

[70] Erickson S.M., Rockwern B., Koltov M., McLean R.M., Medical Practice and Quality Committee of the American College of Physicians (2017). Putting patients first by reducing administrative tasks in health care: A position paper of the American college of physicians. Ann. Intern. Med..

[71] Sinsky C., Colligan L., Li L., Prgomet M., Reynolds S., Goeders L., Westbrook J., Tutty M., Bilke G. (2016). Allocation of physician time in ambulatory practice: A time and motion study in 4 specialties. Ann. Intern. Med..

[72] Accenture Artificial Intelligence is the Future of Growth. https://www.accenture.com/us-en/insight-artificial-intelligence-future-growth.

[73] Bresnick J. Amazon Takes on Unstructured EHR Data with Machine Learning. NLP. https://healthitanalytics.com/news/amazon-takes-on-unstructured-ehr-data-with-machine-learning-nlp.

[74] Hosny A., Parmar C., Quackenbush J., Schwartz L.H., Aerts H.J.W.L. (2018). Artificial intelligence in radiology. Nat. Rev. Cancer.

[75] Nsoesie E.O. (2018). Evaluating artificial intelligence applications in clinical settings. JAMA Netw. Open.

[76] Nundy S., Hodgkins M.L. (2018). The application of AI to Augment Physicians and Reduce Burnout. Health Affairs Blog. https://www.healthaffairs.org/do/10.1377/forefront.20180914.711688/.

[77] AiCure NIH Expects AiCure Technologies’s New Adherence Monitoring Platform to Have “a Significant Impact… [and] Widespread Application in Research and in Care”. Press Release. https://aicure.com/news/nih-expects-aicure-technologiess-new-adherence-monitoring-platform-to-have-a-significant-impact-and-widespread-application-in-research-and-in-care/.

[78] Wartman S., Combs C. (2018). Medical education must move from the information age to the age of artificial intelligence. Acad. Med..

[79] Nuffield Council on Bioethics Bioethics Briefing Note. Artificial Intelligence (AI) in Healthcare and Research. http://nuffieldbioethics.org/project/briefing-notes/artificial-intelligence-ai-healthcare-research.

[80] Davenport T.H., Dreyer K. (2018). AI Will Change Radiology, but It Won’t Replace Radiologists. Harv. Bus. Rev..

[81] https://becominghuman.ai/the-ethics-of-artificial-intelligence-in-healthcare-5fe148427764.

[82] World Health Organisation (2021). WHO Issues First Global Report on Artificial Intelligence (AI) in Health and Six Guiding Principles for Its Design and Use. https://www.who.int/news/item/28-06-2021-who-issues-first-global-report-on-ai-in-health-and-six-guiding-principles-for-its-design-and-use.

[83] Murdoch B. (2021). Privacy and artificial intelligence: Challenges for protecting health information in a new era. BMC Med. Ethics.

[84] Iacobucci G. (2017). Patient data were shared with Google on an “inappropriate legal basis”, says NHS data guardian. BMJ.

[85] More Y.M. (2019). Disaster management using artificial intelligence. J. Xian Uni. Agric. Technol..

[86] Saravi S., Kalawsky R., Joannou D., Casado M.R., Fu G., Meng F. (2019). Use of artificial intelligence to improve resilience and preparedness against adverse food events. Water.

[87] Sakurai M., Murayama Y. (2019). Information technologies and disaster management-benefits and issues. Prog. Disas. Sci..

